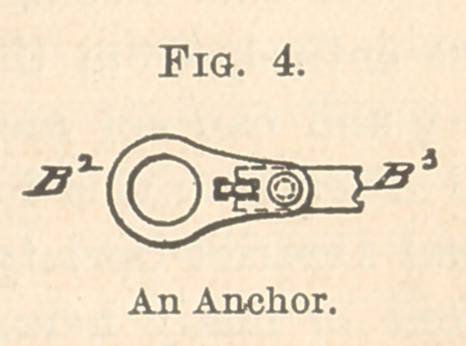# Some of the Causes of Deterioration of Vital Energies of Dentists

**Published:** 1894-05

**Authors:** J. N. Farrar

**Affiliations:** New York City


THE
### International Dental Journal.

VOL. XV. MAY, 1894. No. 5.

### Original Communications.[1]

### SOME OF THE CAUSES OF DETERIORATION OF VITAL ENERGIES OF DENTISTS.

BY J. N. FARRAR, M.D., D.D.S., NEW YORK CITY.

WHY dentists break down in health and fail to hold their ambition is becoming a serious question in the profession. The indoor life necessary to full practice tends to reduction of stamina throughout the entire system, a condition that would not take place from an open-air occupation, such as that of the physician. Especially is this true of the dentist who stands over a badly-formed chair, in a north light, deprived of direct sunlight from morning until evening. He becomes fatigued from long standing and pale from the same cause that makes a plant lose its proper hue when kept in shade. Fatigue, even from moderate work, will result from the weakness caused by a life in shade, and loss of interest in business is the consequence of the fatigue. But, like a horse in a tread-mill, the dentist in "full practice" knows no rest, nor does he feel real enjoyment. Whether well or unwell, he must work on, day after day, month after month, year after year.

If dentists who have no natural weakness in their systems

[1] The editor and publishers are not responsible for the views of authors of papers published in this department, nor for any claim to novelty, or otherwise, that may be made by them. No papers will be received for this department that have appeared in any other journal published in the country.

feel the effect of these strains, those who have tendencies to physical defects, especially in the neck, back, knees, and ankles, must suffer keenly from the long periods of standing. As many know, from experience, who have stood in a crowded house during an opera performance (because there were no seats to be had), the fatigue soon becomes excessively painful, yet the dentist stands for a longer time every day in a worse position, bent over his chair. He may fancy that because he does not feel the fatigue at the time, his mind being absorbed in his operation, his system does not suffer as much as it did at the opera, but the strain upon the system is far greater. Indeed, so great that at the close of the day he often succumbs from general exhaustion, in body and brain.

While the dentist is operating, perhaps there are no parts of the body in which he feels such injuries more than in and about the cervical and lumbar regions along the spinal column. Pain is felt not only in and between the vertebræ, strained and pinched by curving of the column for long periods of time, but also in the erecti muscles about the column, all of which, after a few years, cause that persistent, painful tiresomeness, so well known to nearly every steady worker at the chair, and which is not experienced by the mechanical dentist.

In a negative way this error of life is made evident to him by comparative freedom from such pain and exhaustion on Sundays, when he rests from his labors and sits most of the time. A still stronger evidence is shown by the improvement in the entire system resulting from a long summer vacation, spent in out-door recreations. I speak of long vacations, because *short* ones are often more destructive than beneficial. For years, during my vacations, when after the first few days the strain from routine of business had become relaxed, I was obliged to keep my bed for a considerable part of each day for the first two weeks. When I attempted to take a walk at this time, I could go but a few rods, but after reaching the depths of prostration, and recuperation set in, walks became less and less tedious, until they were a pleasure.

Besides the pains in the neck and other parts of the spine from persistent excessive bending and twisting of the vertebral column, there are those from weakened digestive organs, resulting partly from continuous cramping by stooping and partly from gravitation acting upon the weakened tissues sustaining the organs. The liver suffers not only from pressure by this stooping, but from the twist of the body. The bowels by it also become sluggish, leading ofttimes to clogging and impaction in the region of the cæcum and the sigmoid flexure, causing by reaction loss of appetite and stupefying of mental energy.

If the dentist runs his burring engine himself, he is liable to add to these weaknesses the so-called "sewing-machine trouble," a result of excessive vibratory action of the psoas muscles. If with these ills he be also affected with the "dentist's leg" (as it is called in England), another result of general tissue weakness, there surely is but little left for the dentist to feel happy over.

The question, "What ought we to do to prevent or to correct the evils?" is easier asked than answered, and easier answered than to carry out the proper way of living to avoid them. One person of limited practice suggests "two hours" spent at dinner; another, "an hour or two of horseback-ride or carriage-ride every day;" another, "a half-holiday once a week." All these are beneficial, if it were possible to carry them out. While the leisure dentist may find time for daylight recreation, it would be difficult, if not impossible, for those who are overrun with professional duties. Still, it needs no argument to prove that daytime recreation out of doors is important. But direct sunlight into the operating-room is of very great value, and goes a long way to prevent this deterioration of body. I have tried all lights, and, having had varied experiences carefully recorded, I could dwell upon the great benefit of direct light, but it would not be strictly relevant to the main object of this paper,—*i.e.*, the saving of energy by sitting.

Probably one of the first wrongs to abolish is self-running of the dental engine. This naturally leads to the highly important essential, a chair assistant, who can help, and thereby save the energies of the operator in many ways. Indeed, I regard an assistant of so great value that for thirty years I have had such help, and for many years I have employed two, and generally three.

As before mentioned, the operating-chair has much to do with the health. Great effort has been made to perfect these chairs, but notwithstanding progress in this direction, there is a great deal more to be done before perfection will be accomplished, and some of this must be through a return to discarded parts that are old. Most of the head-rests now made are not only too rickety, but are uncomfortable to both patient and operator, and some of them are ridiculously absurd. If the head-rest is of a kind that requires the operator to stoop or twist himself considerably to get at his work, it is dangerous. I feel confident that my own health has suffered irreparably, in years gone by, from working at a chair that was improperly constructed, and I am sure that many dentists have found an early grave from the same cause. Stooping causes injury to the lungs as well as weakness of the spine and digestive organs. The chair should be so constructed that it will not be necessary for the dentist to stoop or to twist himself much of the time. Who will give us a perfect chair?

A great saving of vital energy is gained by sitting a part of the time during lengthy operations. Of seats for this purpose there are several kinds; one is independent of the operating-chair, another is connected with it, and there is a third that is connected with the wall of the room. The "Lyon's Seat" and the shoemaker's chair are examples of the first, and the dry-goods counter rickrack seat is an example of the last,—the kind connected with the wall.

I generally stand while operating, but have always had a seat to use if I desired. But until 1884 I used one that was indepen-

Fig. 1.

Farrar's Seats for the Operator and Assistant.

dent of the operating-chair, a light, high piano-stool. Since that time I have used one connected with the chair-shaft. (See Fig. 2.) This seat allows me freer action than the independent seat, because it is never in my way, and is easily pushed aside—swung behind the operating-chair—when I have no further use for it. When using it, I generally prefer to stand partly on the right leg, with the left thrown over the seat in such a way as to support most of my weight on the seat. The right leg in contact with the floor enables me to easily move back and forth between the patient and the instrument-table.

Fig. 1 represents a plan of a seat that I devised, which would supply needs of this kind. In this plan there are two seats,—one, *B*, for the dentist, and the other, *D*, for the assistant. These seats are, however, more elaborate than is necessary, unless every convenience is desired. On my chair I have as yet only the seat indicated by $B^1$, Fig. 2. This and the other figures show how the different parts are related. Corresponding letters indicate corresponding parts. This figure, an elevation partly in section, repre-

Fig. 2.

The Operator's Seat.

senting the base of an ordinary dental chair and the operator's seat, shows the relation of the anchors $B^2$, $B^2$ with the shaft $B^1$, and the relation of these parts with the crane ($B^3$) supporting the operator's

Fig. 3.

Anchors as applied.

Fig. 4.

An Anchor.

seat. The several *C*'s represent parts for adjusting by the foot the distance of the seat from the chair.

Fig. 3 represents a side-view of the anchors $B^2$ and their supporting collars, $B^4$, encircling the shaft. These collars are made stationary to the shaft by impinging bolts, $B^9$.

Fig. 4 represents a top view of one of the anchors, $B^2$, for holding the end of the crane, on the other end of which is the seat. The smaller auxiliaries are for fastening the adjustments of the cranes.

These seats and their various parts are somewhat expensive, but simpler ones, constructed on the same general principles, leaving out the foot-adjustments, are not very expensive. Any blacksmith having the seat, screw, and nut of a piano-stool, can easily construct one that will serve the purpose.

## Figures and Tables

**Fig. 1. f1:**
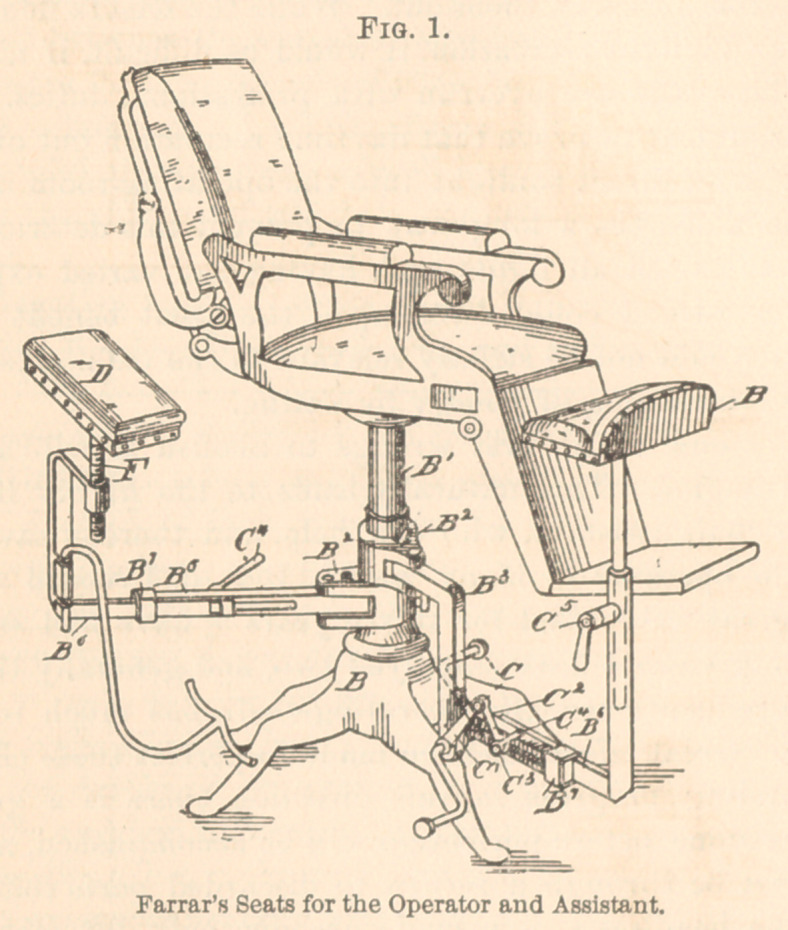


**Fig. 2. f2:**
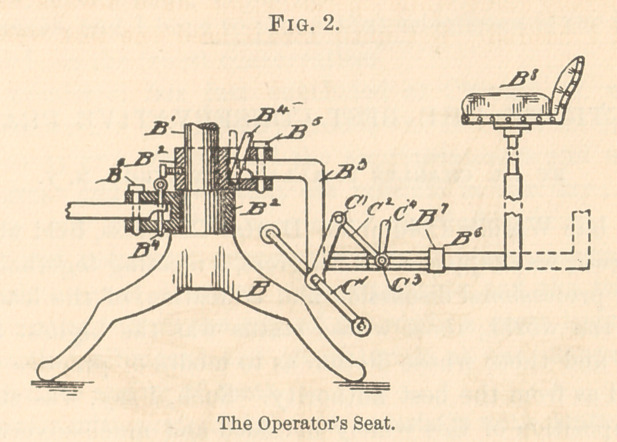


**Fig. 3. f3:**
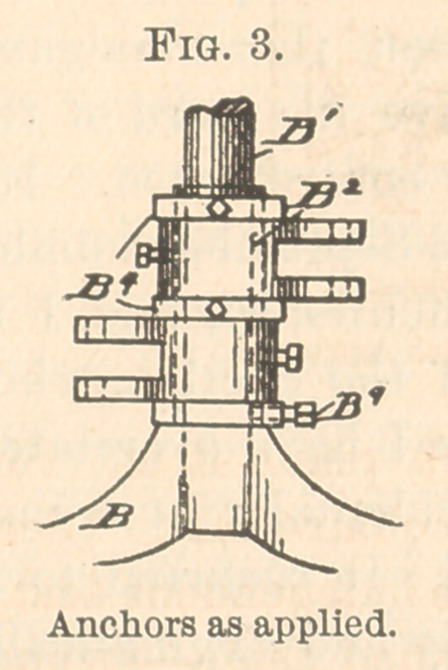


**Fig. 4. f4:**